# Valedictory

**Published:** 1867-03

**Authors:** 


### EDITORIAL.

VALEDICTORY.—Circumstances having arisen, which render a change in the editorial management of the *Journal* desirable, we once more resume our position in the immortal army of subscribers to the medical literature of the land. With naught but pleasant memories of the duties and the relations which pertained to our official term, and with the best of wishes for the prosperity of our successor, we surrender the keys of the *sanctum sanctorum* of the CHICAGO MEDICAL JOURNAL.

